# Reframing advisory as leadership to promote equity in trauma-informed community-engaged research: A case study of Yolo County, CA

**DOI:** 10.1017/cts.2026.10693

**Published:** 2026-02-10

**Authors:** Leigh Ann Simmons, Jasmine Cuellar, Jennifer Phipps

**Affiliations:** 1 Betty Irene Moore School of Nursing | Clinical and Translational Science Center, University of California Davis, USA; 2 Betty Irene Moore School of Nursing, University of California Davis, USA

**Keywords:** Community-engaged research, health equity, trauma-informed care, power-sharing, community-academic partnerships

## Abstract

**Background:**

Community engagement that emphasizes shared leadership is essential in clinical and translational science, and language, naming, and framing have the potential to shape power dynamics. This study explored how renaming and restructuring a Community Advisory Board (CAB) into a Community Leadership Board (CLB) could strengthen a trauma-informed network of care (TINoC) by elevating community power, cultural responsiveness, and equitable participation.

**Methods:**

Guided by the Trauma and Resilience Informed Research Principles and Practice(TRIRPP) framework, we established a paid CLB in Yolo County, California, composed of six individuals who identified as members of groups underrepresented in science. We reviewed timesheets and TINoC products and conducted an inductive thematic analysis of meeting minutes to determine the CLB’s main areas of influence.

**Results:**

The CLB met 25 times over two years, provided iterative feedback on more than a dozen educational materials, clinical workflows, trauma-informed trainings, and communication strategies, and co-presented at community meetings. Eight recurring areas of influence were identified: trauma-informed ACE screening, accessibility, workflow feasibility, community- and patient-centered feedback, health communication, participant compensation, engagement, and post-screening navigation. CLB members highlighted gaps not identified by the academic and community members of the TINoC, including translation accuracy, time allowed for ACE screening, and ensuring voluntary patient participation.

**Conclusions:**

Renaming the CLB as a “leadership” body signaled a shift in accountability, deepened engagement, and underscored how naming practices can drive more equitable translational research. Virtual-only meetings potentially limited the representativeness of the CLB; however, results suggest naming is a critical component of trauma-informed community-engaged research(CEnR).

## Introduction

Community engagement that centers shared leadership is essential to clinical and translational science. Trauma-informed care (TIC) is both an organizational philosophy and a clinical approach that recognizes how past and present experiences shape behavior and health, and emphasizes safety, trust, peer support, and collaborative partnerships with patients and families [1–3]. By restoring decision-making power to patients as experts in their own bodies and lived experiences, TIC supports culturally responsive, contextually relevant care [4,5]. Trauma-informed networks of care (TINoCs) extend these principles by uniting health systems, educational institutions, community-based organizations (CBOs), and social service agencies to collaboratively prevent, identify, and mitigate the effects of Adverse Childhood Experiences (ACEs) [6–9].

Meaningful community engagement is a cornerstone of effective TINoCs [3]. Traditionally, community advisory boards (CABs) have served as a primary mechanism for incorporating community perspectives into organizational agendas. However, *advisory* commonly denotes “the power to recommend actions but not to take action to enforce them [10],” and empirical evidence demonstrates wide variability in CAB influence, with members frequently reporting tokenism and limited power-sharing [11–13]. In medical and social services settings, contexts already marked by structural power imbalances, such limitations are particularly consequential. Language that minimizes authority or reinforces hierarchical roles can undermine trust and exacerbate disparities, particularly among individuals with lived experiences of trauma [3].

Prior work in community-based participatory research (CBPR) and community-engaged research (CEnR) highlights the central role of power, equity, and meaningful engagement in shaping research priorities, processes, and outcomes [3,6,11–17]. When community members are positioned primarily as advisors, their influence on decision-making is often constrained. In one study, only 33.3% of CAB members reported that research teams promoted mutually beneficial relationships, and fewer than half reported adequate power-sharing [12]. In contrast, structures that provide genuine leadership authority, shared governance, and shared decision-making are associated with higher member satisfaction (up to 98%), expanded professional and social networks (76%), increased knowledge (84%), and improved research relevance in approximately 90% of projects [6,16,17]. Collectively, these findings suggest that explicitly structuring community boards as leaders, rather than limiting members to advisory roles, can strengthen engagement, empowerment, and equity in CEnR.

**Figure 1.**
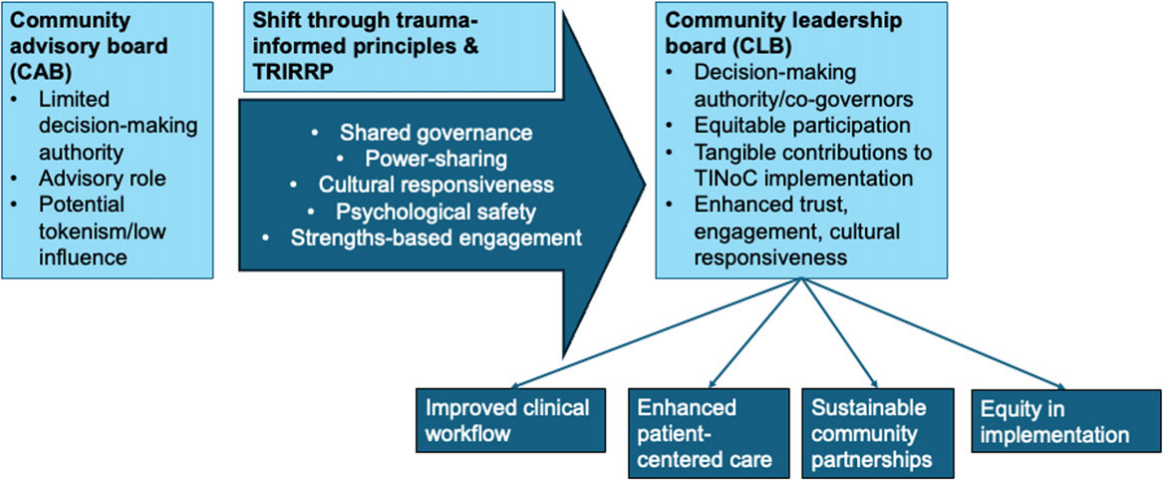
Conceptual framework for transitioning from a Community Advisory Board (CAB) to a Community Leadership Board (CLB) within the Trauma-Informed Network of Care (TINoC). The framework highlights shifts in governance (direction, alignment, commitment), decision-making authority, accountability mechanisms, and trauma-informed facilitation. CAB = Community Advisory Board; CLB = Community Leadership Board; TINoC = Trauma-Informed Network of Care; ACEs = adverse childhood experiences; TRIRPP = Trauma and Resilience Informed Research Principles and Practice.

Language is a crucial mechanism through which power is distributed in community-engaged work [14,18]. In trauma-informed contexts, naming practices are not merely semantic. Rather, they shape expectations, signal authority, and influence whether individuals feel safe to participate fully [3]. Leadership, as defined by the Center for Creative Leadership, is “a social process that enables individuals to work together to achieve results that they could never achieve working alone [19].” Drawing on this framework, we intentionally established a Community Leadership Board (CLB), rather than a CAB, within our Yolo County TINoC to formalize shared governance, accountability, and collective ownership of decision-making. This framing positions community members not as peripheral consultants, but as essential co-governors responsible for guiding priorities, approving materials and workflows, and ensuring that lived experiences of trauma directly inform TIC strategies. Figure 1 illustrates this conceptual shift from advisory to leadership-based governance.

Consistent with trauma-informed principles, the CLB was designed not only to confer authority but also to protect psychological safety. Because many CLB members received services from organizations within the TINoC, governance structures were intentionally developed to minimize perceived risks of retaliation and to support open, candid feedback. These design features reflect an understanding that leadership without safety can inadvertently reproduce harm, even when participation is nominally empowered.

This manuscript advances the field by operationalizing these principles using the Trauma and Resilience Informed Research Principles and Practice (TRIRPP) framework [4]. We describe the formation and activities of a paid CLB, detail its influence on trauma-informed ACE screening, accessibility, workflow feasibility, health communication, compensation practices, engagement strategies, and resource navigation, and present CLB members’ reflections on leadership identity. We further examine how intentional naming and governance structures reshaped power dynamics within a TINoC, offering a replicable model for embedding authentic community leadership across TIC initiatives and beyond.

## Materials and methods

### Setting and TINoC partners

We applied a CEnR approach to implement a TINoC in Yolo County, CA, through an ACEs Aware-funded Initiative. The TINoC included seven core contributors and four supporting community-based organizations (CBOs) representing health care, education, family services, and community resilience sectors. UC Davis served as the administrative lead, alongside two Federally Qualified Health Centers providing comprehensive care to low-income and farmworker populations. First 5 Yolo and Yolo County Children’s Alliance supported family health and early childhood development. The Resilient Yolo collaborative represented 25 county agencies united by a shared goal of strengthening resilience through ACEs education. Unite Us served as the referral hub, enabling cross-sector coordination of services. Additional partners, Stanford Sierra Youth & Families, Origins Training and Consulting, Creative Behavior Systems, and the Redwood Community Health Coalition, supported behavioral health services, workforce development, trauma-informed training, and regional data sharing.

### Community leadership board formation and safeguards

We assembled a paid CLB to reflect the diversity of Yolo County residents receiving services and to ensure that cultural and contextual perspectives informed network strategies. Each partner organization nominated an actively engaged service user, ensuring representation across racial and ethnic backgrounds, ages, gender identities, and primary languages. Prospective members received a position description outlining their responsibilities: (1) serving as the eyes, ears, and voices of the community by contributing lived experience and expertise as leadership team members; (2) committing one to two hours every other week, primarily through Zoom meetings facilitated by a bilingual (English/Spanish) community engagement specialist; (3) participating in all TINoC activities, including co-design, iterative feedback, and final approval of educational and outreach materials and implementation strategies prior to network-wide dissemination; (4) retaining the right to decline requests at any time without providing a reason; and (5) receiving financial compensation for all participation, including meeting time, material review, clinic visits, and any related activities.

Because many CLB members also received services from participating organizations, concerns arose that negative feedback could affect their care or interactions with providers. To mitigate this risk, the community engagement specialist facilitated all CLB meetings, synthesized member feedback, and communicated it to researchers and partner organizations. This structure positioned the specialist as a bridge between the CLB members and the TINoC partners, and it supported open, candid input. These safeguards enabled CLB members to share perspectives, ideas, and decisions freely, whether positive or critical.

### Data sources and analysis

We summarized participation metrics (e.g., the frequency of product and process reviews) and analyzed two years of CLB meeting minutes. After each meeting, the community engagement specialist transcribed detailed handwritten notes and stored them securely online. The minutes documented agenda items, discussion topics, implementation milestones, and CLB feedback on project deliverables. We treated the transcribed minutes as qualitative data and conducted an inductive thematic analysis to identify recurring themes, priorities, and feedback that informed TINoC processes and outcomes.

### Reflexivity and member validation

At least two research assistants who were not involved in CLB activities independently reviewed the meeting minutes and generated preliminary themes. To support inter-rater reliability, each reviewer conducted independent coding before convening with the broader research team (five reviewers total) to compare findings. Through discussion, the team integrated overlapping themes and reached consensus, prioritizing themes that appeared consistently across reviewers. The community engagement specialist then contextualized the themes based on her role as facilitator. To address potential bias associated with this dual role, we relied on independent initial coding and collective interpretation. Group consensus ensured that multiple perspectives shaped final interpretations.

Consistent with trauma-informed and equity-centered principles, we invited CLB members to serve as co-authors; however, all members preferred an acknowledgment. In response to journal revision requests, we re-engaged the CLB to reflect on the use of the terms *“advisory”* versus “*leadership*” and to assess whether “*leadership”* accurately represented their experiences. CLB members reviewed and validated all themes to ensure accurate representation.

### Analytic framework

We organized results using the TRIRPP framework [4]. This framework comprises eight principles designed to improve the inclusion and research experience of disadvantaged populations in health and social care settings: (1) actively seeking participation from disenfranchized individuals and groups; (2) addressing deprivation and health inequities through a social justice lens; (3) framing researcher-participant relationship as relational rather than authoritative; (4) promoting choice and agency to empower individuals and communities; (5) emphasizing strengths and resilience; (6) minimizing re-traumatization; (7) recognizing the impact of trauma and adversity across participants; and (8) promoting cultural competence and safety. The TRIRPP framework guided both our analytic approach and presentation of results.

## Results

### Participation

Over two years, CLB members attended 25 meetings totaling approximately 30 hours and consistently met or exceeded the established quorum of at least four of six members (Table 1). Members provided iterative feedback across a wide range of activities and products, in some cases requiring up to nine rounds of revision before finalization (Table 2). Altogether, their contributions directly informed the development of one clinical workflow process, two trauma-informed trainings, and eight products: one animation video, four mini-documentaries, two patient-facing handouts, and one digital ACE screening application.

**Table 1.** Demographic characteristics of Community Leadership Board (CLB) members

*Gender*	*Race/ethnicity*	*Family status*	*Age*
Female	Hispanic/Latine	Parent	44
Female	Hispanic/Latine	Parent	42
Female	White	No children	60
Female	White	Foster/adopted parent	28
Female	Hispanic/Latine	Parent	41
Trans and nonbinary	Black	Parent	35
Female	Hispanic/Latine	Parent	38

At any given time, six individuals served on the CLB; a seventh replaced a member who withdrew due to family obligations. Variables include age, gender identity, race/ethnicity, and family status. CLB = Community Leadership Board.

**Table 2.** Community Leadership Board (CLB) engagement across key themes and deliverables

*Theme(s)*	*Activity*	*Number of CLB contributions*	*Products influenced*	*Iterations/notes*
Trauma-informed ACE screening process; accessibility considerations	ACEs educational video	8 reviews	1 animation video	6+ iterative revisions
Enhancing health communication strategies	Documentary mini-series	9 reviews	4 videos	Storytelling & subtitles guided by CLB
Clinical feasibility and workflow integration; patient experience and post-screening resource navigation	Clinic walkthroughs	8 sessions	Workflow integration	Identified gaps, recommended patient support
Community and patient-centered feedback; enhancing health communication strategies; strengthening trauma-informed community engagement	Community events	3 sessions	Resilient Yolo Summit	Planning, panel co-facilitation, giveaways
Enhancing health communications strategies; community and patient-centered feedback	Patient-facing materials	2 reviews	Stress Busters handouts	Content simplified, localized, trauma-informed
Trauma-informed ACE screening process; accessibility considerations; patient experience and post-screening resource navigation	ACEs screening app	1 review	App design	User-friendly, multilingual, optional score sharing
Trauma-informed ACE screening process	Trauma-informed training	2 formats	62 staff trained	Online modules + in-person “train-the-trainer”

Summary of review cycles, products influenced (clinical workflow; trauma-informed trainings; animation and mini-documentary videos; patient-facing handouts; ACE screening app), and meeting participation. CLB = Community Leadership Board; ACEs = Adverse Childhood Experiences; TINoC = Trauma-Informed Network of Care.

**Figure 2.**
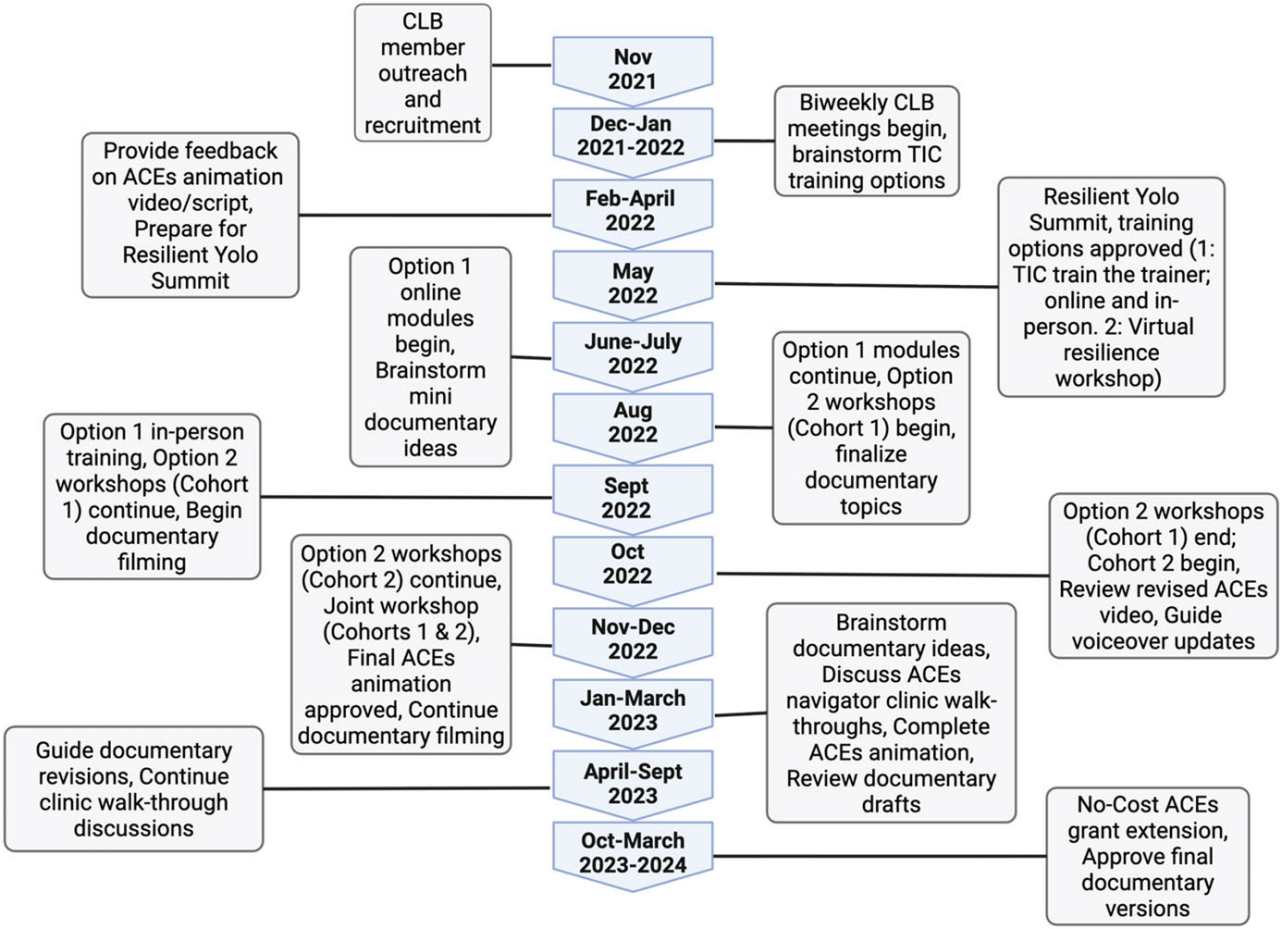
Implementation timeline of the Community Leadership Board (CLB) across the two-year study period. Major milestones include recruitment and onboarding, meeting cadence, iterative reviews of educational materials and clinical workflows, trauma-informed trainings, co-presentations at community events, and network-wide dissemination. CLB = Community Leadership Board; TINoC = Trauma-Informed Network of Care; ACEs = Adverse Childhood Experiences; CBOs = Community-Based Organizations.

A total of 62 clinical and non-clinical staff completed TIC training, including 16 participants in the Train-the-Trainer series (online and in-person), and 46 participants in the Virtual Resilience Workshop Series (30 staff in Cohort 1 and 16 in Cohort 2). Together, these activities reflect the CLB’s direct role in embedding trauma-informed approaches across clinical and community-based settings (Figure 2).

### CLB member reflections on leadership identity

CLB members consistently expressed that the term *leadership* more accurately captured their level of involvement, accountability, and influence in decision-making. One member emphasized: *“I felt from the very beginning of [the] project that our enlightened opinions would be listened to and our recommendations followed, absent some good reason. I was accorded the respect due to being a leader, as opposed to a person or entity that can be followed or not, depending on the situation. Too often agencies will mouth the words and then avoid the hard work of implementation, and we end up with nothing changing. Change is never easy and in old attitudes one always finds resistance. If [the] CLB and its [partners] can work together with recommendations being recognized as required, [this] is where the words advisory to leadership make a difference. I was employed by many government agencies (large bureaucracy), and change is implemented not suggested.”* Other members echoed this sentiment, highlighting that terminology shaped how their contributions were received and acted upon. As one noted, *“Leadership is good and more formal, which shows that it is more to create change.”* Another added: *“I prefer leadership because I feel taken more seriously and feel that whatever we request or suggest will be acted upon since we all are leaders. Advisory is also good, but it is more just suggestions, and not as strong as leadership, [and] it would be downplaying everybody’s effort.”*

### Qualitative results

Eight broad themes emerged from the qualitative analysis, illustrating the CLB’s leadership in shaping equitable, culturally responsive TIC. These themes are summarized below. Tables 3 and 4 map each theme to the TRIRPP framework principles and associated outcomes.

**Table 3.** Alignment of CLB influence with the Trauma and Resilience Informed Research Principles and Practice (TRIRPP) framework

		TRIRPP principles							
		*Take active steps to seek participation from disenfranchized groups and individuals*	*Unite with social justice; tackling deprivation and health inequities*	*Frame the researcher–participant relationship as relational*	*Empower individuals and communities through choice and agency*	*Emphasize strenghts and resilience*	*Minimize re-traumatization*	*Recognize potential impact of trauma and adversity in all participants*	*Strive to be culturally competent and promote safety*
**CLB Themes**	Trauma-informed ACEs screening processes				Give option to educate about ACEs before screening; provide the choice to share final ACE score		Do not mandate ACEs screening for all	Provide resource referrals even if patient/parent chooses not to screen for ACEs	
	Accessibility considerations	Designing educational video to be inclusive	Creation of an educational video to save providers time teaching patients about ACEs						Hire native speakers in the primary languages in Yolo County for videos
	Clinical feasibility and workflow integration						Provide trauma-informed training to all members of each partner organization		
	Community and patient-centered feedback					CLB members identified barriers to accessing trauma-informed care based on their lived experiences			Allow patients to review ACEs video in private
	Enhancing health communication strategies	Make “stress buster” fliers accessible and inclusive	Develop mini-documentaries to highlight the role of ACE screening in primary care and the impact of trauma-informed approaches						
	Compensation and support for participants	CLB suggested giveaway items (e.g., diapers, self-care items) to community members attending community-building programs	Provide CLB members with financial compensation	All participation from CLB members is voluntary; all feedback is considered important (rather than a suggestion)	CLB members provide feedback on all community engagement and academic activities of the TINoC	Provide leadership training to CLB members			
	Strengthening trauma-informed community engagement	Targeted outreach to historically marginalized communities		CLB members participated in the trauma-informed training they approved for the TINoC					Create materials that are culturally inclusive
	Patient experience and post-screening resource navigation	CLB members suggested ways to improve the ACE screening process						CLB members walked through a sample ACE screening to evaluate patient experience	

Mapping of TRIRPP principles to CLB contributions, illustrative examples, and related outcomes. CLB = Community Leadership Board; TRIRPP = Trauma and Resilience Informed Research Principles and Practice; TINoC = Trauma-Informed Network of Care; ACEs = Adverse Childhood Experiences.

**Table 4.** Recommendations for integrating Community Leadership Boards (CLBs) into public health interventions based on the TRIRPP framework

		Recommendations for integrating CLBs into public health interventions based on TRIRPP principles							
		*Take active steps to seek participation from disenfranchized groups and individuals*	*Unite with social justice; tackling deprivation and health inequities*	*Frame the researcher–participant relationship as relational*	*Empower individuals and communities through choice and agency*	*Emphasize strenghts and resilience*	*Minimize re-traumatization*	*Recognize potential impact of trauma and adversity in all participants*	*Strive to be culturally competent and promote safety*
**Domain**	Equitable partnerships & governance	Frame community-led groups as leadership boards rather than advisory boards to emphasize power-sharing		Frame community-led groups as leadership boards rather than advisory boards to emphasize power-sharing	Establish clear processes for shared decision-making and co-authorship on outputs				
	Compensation & support	Provide financial remuneration to acknowledge time and expertise			Allow flexible levels of participation based on member comfort		Allow flexible levels of participation based on member comfort		Designate a community engagement specialist to facilitate communication and ensure psychological safety
	Product & intervention development	Engage CLB members in co-developing, testing, and approving materials		Engage CLB members in co-developing, testing, and approving materials					Use iterative feedback loops with multiple revisions to ensure accuracy and cultural responsiveness
	Equity & inclusivity	Recruit CLB members intentionally from historically marginalized communities (HMCs)	Incorporate CLB input to identify and address exclusionary or biased practices					Incorporate CLB input to identify and address exclusionary or biased practices	Ensure materials are accessible (e.g., multiple languages, diverse representations)
	Empowerment & sustainability		Empower CLB members to advocate for community health needs through leadership opportunities	Build skills and provide recognition (e.g., authorship, public presentations) to strengthen sustainability		Position CLB members as lived-experience experts in community events and education			
	Trauma-informed practices					Evaluate processes through a strengths-based lens	Minimize re-traumatization by embedding TIC principles in all activities	Provide TIC training for both CLB members and staff Use trigger warnings and preserve confidentiality in meeting notes	
	Cultural responsiveness & patient-centered care			Promote trust, respect, and agency in clinical and community interactions		Recognize patients as experts of their own experiences			Integrate CLB insights to ensure culturally competent, patient-centered approaches

Target settings, responsible parties, required resources, and anticipated outcomes, with attention to historically marginalized communities. CLB = Community Leadership Board; TRIRPP = Trauma and Resilience Informed Research Principles and Practice; HMCs = historically marginalized communities.

#### Trauma-informed ACE screening processes

In line with emerging California priorities [20], a central tenet of the TINoC was the integration of ACE screening and referral to buffering services within primary care workflows at participating federally qualified health centers. The CLB guided the design and implementation of this workflow by identifying key ethical and practical considerations.

Based on their input, patients first received education about ACEs, their health impacts, and the rationale for screening in primary care through an original animated video, which included a content warning in its final version. CLB members raised concerns about universal (“mandated”) screening, leading to recommendations for age-appropriate, separate consent or assent for patients and parents rather than automatic family-wide screening. Patients who consented to screening could also choose whether to share their ACE score with their provider.

In the initial design, ACE screening was to be used to inform referrals to buffering support services. However, the CLB raised concerns about access to referrals for individuals who declined screening. In response, we redesigned the workflow to include a menu of available social services, allowing patients and their parents to express interest in possible referrals to these services regardless of screening status. Throughout this process, CLB members highlighted the importance of minimizing re-traumatization, addressing mandated reporting concerns, and creating safe screening environments (e.g., dedicated spaces and attention to caregiver and child comfort). Partner clinics also adopted these recommendations in the final workflow.

#### Accessibility considerations

To reduce provider burden, TINoC partners delivered ACEs education and screening through a mobile application and an animated educational video, both developed in collaboration with CLB members. The tools included language-specific subtitles, and clinics provided earphones for private viewing. CLB members shaped both products through iterative feedback and usability testing, emphasizing visually engaging, multilingual materials that reflected Yolo County’s diversity.

In response, the project hired native-speaking actors for the six most spoken non-English languages in the county (Spanish, Russian, Punjabi, Mandarin Chinese, Hmong, and Urdu), rather than relying solely on subtitles. CLB members affirmed that this approach enhanced cultural resonance and relatability. Patients and parents could also complete ACE screening using paper forms instead of the digital survey.

The CLB identified early versions of the video as overly distressing and directed revisions to enhance trauma-informed delivery. They flagged potentially triggering elements, including a lengthy enumeration of ACEs, somber background music, and an intense depiction of parental conflict. At their recommendation, the production team removed pill bottles from scenes depicting addiction and revised the closing to include a caring adult holding a child’s hand, conveying safety and healing. This iterative process extended the production timeline to more than three times the initial estimate.

#### Clinical feasibility and workflow integration

Recognizing clinical time constraints, CLB members guided integration of ACE screening and social service referrals into existing workflows. Recommendations included pre-visit educational materials and trauma-informed communication training for clinicians. While these approaches aligned with initial TINoC plans, CLB members provided insight into community centered implementation. As a result, the project delivered trauma-informed training approved by CLB members for TINoC providers. CLB members also participated in these trainings, increasing their leadership capacity in TIC.

#### Extending community and patient-centered feedback

As TINoC partners increasingly recognized the CLB’s value, its influence extended beyond the ACEs project. CLB members co-led planning for the 2022 Resilient Yolo Summit, which highlighted community-based programs and TINoC progress, including trauma-informed services. They curated family-centered self-care giveaways to promote engagement and co-facilitated a panel discussion, sharing perspectives as community experts and TINoC co-leaders. CLB members shaped outreach materials for First 5 Yolo’s Welcome Baby: Road to Resilience program by directing revisions to improve clarity, transparency, and inclusivity. In addition, CLB members reviewed grant application materials prior to submission for the National Institutes of Health Research to Advance Connected and Community Health (ReACH) Equity T32 predoctoral training program at the Betty Irene Moore School of Nursing at UC Davis (T32NR021294), and currently participate in annual graduate student fellow selection.

#### Enhancing health communication strategies

CLB members strengthened patient- and client-facing materials by simplifying language and enhancing cultural relevance. For example, they revised the ACEs Aware “Stress Buster” handouts by reducing text and incorporating local resources, resulting in concise Yolo County-specific materials. Beyond the educational pre-screening video, CLB members guided production of mini-documentaries showcasing ACE screening in primary care and trauma-informed practices across healthcare and education settings. They emphasized conversational dialog, natural pacing, and the use of subtitles to increase accessibility and engagement.

#### Compensation and support for CLB members

CLB members received compensation of $30 per hour for all activities, including meetings, material review, communications, trainings, and events. Members completed monthly activity trackers to ensure accurate payment. This approach aligned with TINoC values by recognizing community expertise as equivalent to professional expertise, fostering equity and inclusion. Participation also expanded leadership skills and provided paid work experience.

#### Strengthening trauma-informed community engagement

CLB members guided outreach to underrepresented populations, including LGBTQ + communities, Black/African American residents, and individuals without immigration status. In developing four mini-documentaries, they prioritized intersectional and inclusive storytelling. The resulting videos addressed: (1) the health impacts of ACEs; (2) the purpose and value of ACE screening; (3) a CLB member’s lived experience with ACEs; and (4) trauma-informed practices in community settings such as schools. These videos were shared through the TINoC and are publicly available on YouTube.

CLB members emphasized the value of trauma-informed training for both network staff and community leaders. In response, TIC experts delivered two training programs focused on toxic stress, resilience, and trauma-informed approaches. The first included an online component and a one-day in-person Train-the-Trainer session tailored to participating organizations. CLB members who completed the online training reported increased understanding of ACEs and toxic stress in their own lives. The second program centered on resilience and was delivered across two cohorts.

#### Patient experience and post-screening resource navigation

CLB members participated in a clinic walkthrough to evaluate the patient experience of ACE screening at federally qualified health centers. Patient navigators guided CLB members through check-in and screening procedures as if they were patients. This process revealed gaps in workflow and post-screening resources. For example, CLB members noted that patient navigators, who were not licensed behavioral health specialists, needed clear referral pathways for patients who experienced distress after viewing the video or completing the screening. They also identified that there was insufficient time allotted for patients newly learning about ACEs to reflect, review resources, and select referrals. As one CLB member explained, “*The [screening] questions for adults make you question and pause to reflect on one’s life and makes you think about the past. Fifteen to twenty minutes is not enough time for this reflection while doing the ACE screenings. It’s too short of a time to try to help someone when there are so many thoughts and ideas that come to light.*” In response, the CLB recommended extending time with navigators or providers, especially for patients new to ACEs education or those requiring immediate support.

## Discussion and future directions

Equitable partnerships in CEnR are essential for building trust and fostering sustainable relationships with communities [15]. The TRIRPP framework recommends intentional inclusion of disenfranchized groups and a relational approach to researcher-participant dynamics [4]. These relationships are crucial to reducing health disparities because they foster shared decision-making and collaborative action to improve health and resilience [21,22]. Our work contributes to the growing CEnR scholarship by demonstrating how trauma-informed naming and governance structures move beyond theory to practice, redistributing decision-making power and embedding equity within healthcare networks.

By using the TRIRPP framework to structure the CLB and document its contributions to TINoC implementation, this study illustrates how trauma-informed governance can enhance inclusivity, responsiveness, and accountability in community-academic partnerships. We intentionally named the group a “leadership” board rather than an “advisory” board to operationalize shared governance and dismantle hierarchical models in which community boards provide input without authority to enact change [11–13,16,17]. Consistent with prior work, we found that treating community partners as co-leaders increased research relevance, satisfaction, and trust [6,16,17].

Positioning CLB members as co-governors rather than advisors directly addressed structural limitations common in traditional engagement models and ensured community leadership across all phases of trauma-informed implementation. CLB member reflections highlight how both naming and structure influenced power in practice. While power imbalances inevitably exist in community–academic collaborations, CLB members helped minimize these dynamics through intentional design, trauma-informed facilitation, and accountability to community-defined priorities. By grounding leadership authority in lived experience, this approach builds on CBPR and CEnR principles while advancing a trauma-informed model of shared governance.

From the outset, we prioritized reciprocal relationships with CLB members from underrepresented communities. Designating the group as a leadership board formally recognized their authority, while equitable pay and consistent support from a community engagement specialist demonstrated respect for their expertise and time. The specialist protected confidentiality, fostered psychological safety, and mediated feedback between community and institutional partners. CLB members engaged at varying levels, with some serving as livedexperience experts at community events and others co-developing ACEs education or reviewing products iteratively. Across all activities, the TINoC emphasized co-creation rather than consultation, aligning with TRIRPP principles that recognize trauma’s widespread impact and prioritize environments that minimize re-traumatization.

Given the high prevalence of ACEs, recognizing the pervasiveness of trauma was essential. CLB members helped the TINoC move beyond a narrow ACE framework to address structural and intersectional adversities, including racism, discrimination, immigration stress, and disability-related trauma [9,23,24]. By broadening how ACEs were conceptualized, CLB members ensured that interventions reflected the lived realities of those most affected by structural inequities.

CLB members had a substantial impact on project deliverables. Member input shaped trauma-informed ACE screening processes, patient-facing materials, and provider workflows. For instance, members emphasized that universal ACE screening without consent could retraumatize or stigmatize individuals, prompting the TINoC to decouple ACE screening from social service referrals. This approach allowed patients to access resources through choice-driven, non-stigmatizing pathways. CLB members also influenced the format and pacing of educational materials, ensuring visual representation, linguistic accessibility, and alignment with real-world clinic flow. Without this feedback, structural barriers and biases such as linguistic exclusion, cultural incongruence, or time constraints might have gone unrecognized and inadvertently exacerbated disparities.

The CLB model that emerged from this process demonstrates scalability across public health initiatives, including chronic disease prevention, maternal-child health, and mental health promotion. Sustainability requires infrastructure that supports authentic engagement, including dedicated staff, fair compensation, and institutional commitment to shared governance. Embedding CLBs within health systems, rather than treating them as time-limited or grant-dependent, can enhance both sustainability and impact. Institutional and policy-level commitments that allocate resources for community leadership and integrate CLB activities into system priorities are essential for advancing long-term equity.

CLB participation also advanced culturally responsive and patient-centered care, which is strongly associated with improved health outcomes [25–28]. When clinicians engage patients as trusted partners rather than authoritative experts, patients are more likely to feel respected and empowered. CLB members’ lived experiences informed strategies that strengthened this approach within the TINoC, ensuring interventions were relevant, respectful, and grounded in community needs. Their contributions exemplify how trauma-informed, equity-centered governance can transform both research and practice by shifting power, enhancing trust, and creating enduring systems change.

### Limitations

Despite these strengths, this study has limitations. Project initiation coincided with the onset of the COVID-19 pandemic, which constrained recruitment of CLB members to referrals from TINoC partners rather than open community calls. This approach may have narrowed the participant pool and limited representation from underrepresented Yolo County communities most affected by ACEs and toxic stress. All CLB meetings were held virtually due to public health restrictions. While this format enabled continuity, it likely limited opportunities for deeper interpersonal connection, informal dialog, and reflection that occur during in-person engagement. Virtual participation may also have posed barriers for individuals with limited access to stable internet, private space, or technological literacy.

Not all CLB members were initially familiar with ACEs or toxic stress, which led to early confusion regarding some project materials. We addressed this challenge through iterative education and discussion. Despite these constraints, we did not encounter significant barriers in balancing academic and community priorities, primarily because CEnR principles were applied proactively. Although internal deadlines were occasionally extended, these adjustments were anticipated and documented. Compensation equity was maintained across partners, and potential conflicts between academic and community priorities were monitored through transparent processes and regular engagement with CLB members.

Because this work reflects a single regional network and one CLB operating under pandemic-era constraints, the findings may not be generalizable to all contexts. However, the processes described offer a replicable framework for integrating trauma-informed, equity-centered principles into community partnerships. We also acknowledge that power imbalances are inherent in academic-community partnerships. While these dynamics cannot be eliminated, our intentional framing of the CLB as a leadership body represents an ongoing effort to mitigate structural inequities.

## Conclusion

CLB members demonstrated the value of centering lived experience in trauma-informed systems change. Language and naming matter because they shape understanding, authority, and power. The distinction between a CAB and a CLB is substantive, with leadership indicating the genuine co-governance of initiatives with community members. By leveraging CLB member insights, we enhanced accessibility, cultural responsiveness, and patient-centered care while minimizing the risk of re-traumatization.

Future CEnR efforts should prioritize lived experience to ensure that interventions meaningfully respond to community needs. Naming Community Leadership Boards(CLB) accordingly represents a critical first step. By providing a structured framework for establishing leadership boards rather than advisory boards, and by demonstrating their tangible impacts on TINoC implementation, this model can be adapted across diverse geographic and organizational contexts. Translational research teams can apply these principles to strengthen community voice, equity, and shared decision-making across public health and clinical initiatives. Intentional naming, governance structures, and trauma-informed operationalization of CEnR principles can be systematically incorporated to achieve both equitable processes and measurable improvements in outcomes.
